# Competencies and learning outcomes for healthcare professionals in climate change and sustainability: A protocol for a scoping review

**DOI:** 10.12688/hrbopenres.13966.1

**Published:** 2024-10-16

**Authors:** Emer Galvin, Anél Wiese, Niamh Coakley, Deborah Heaphy, Marah Elfghi, Caoimhe O'Brien, Claudia Osborne, Rory Mulcaire, Deirdre Bennett

**Affiliations:** 1 Medical Education Unit, School of Medicine, Brookfield Health Sciences Complex, University College Cork, Cork, County Cork, T12 AK54, Ireland

**Keywords:** climate change; health professions’ education; healthcare professionals; planetary health; environmental sustainability; scoping review

## Abstract

**Rationale:**

The planetary crisis is a serious threat to human health. Healthcare professionals need to be trained to adapt to and mitigate against this crisis. Competencies, curricular frameworks and learning outcomes relating to climate change and sustainability (CC&S) have been proposed for healthcare professionals. A synthesis of these competencies, learning outcomes and frameworks is necessary to identify commonalities and differences, understand the process of their development and highlight areas for future development.

**Objective:**

The objective of this scoping review is to identify and synthesise the evidence on competencies, curricular frameworks and learning outcomes for healthcare professionals in climate change and sustainability.

**Inclusion criteria:**

Sources relating to healthcare professionals and healthcare students, describing competencies, curricular frameworks and learning outcomes in CC&S, will be included. Sources in all healthcare contexts will be included. Sources in the English language, published from 2014 to June 2024 will be considered for inclusion.

**Methods:**

The review will be conducted in line with the Joanna Briggs Institute guidance for scoping reviews. The following electronic databases will be searched: PubMed, Embase, CINAHL, PsycINFO, SocINDEX, Academic Search Complete, Business Source Complete, British Education Index, Australian Education Index, Scopus and ERIC. A search of the grey literature will also be conducted. Two reviewers will independently screen the titles and abstracts and full texts for eligibility. Data extraction will be conducted independently by two reviewers. A narrative summary and tables will be presented. Key stakeholders will be consulted throughout the review.

**Discussion:**

This review will summarise the range of competencies, curricular frameworks and learning outcomes proposed internationally for various healthcare professionals. The findings will be used to inform core competencies for all healthcare professions in CC&S, in addition to highlighting gaps in the literature and areas for future development. The findings will be disseminated at conferences and in a peer-reviewed publication.

**Registration:**

This protocol was registered on 31 ^st^ July 2024 on the Open Science Framework ( https://osf.io/vnx2g).

## Introduction

The planetary crisis, comprising climate change, biodiversity loss, and pollution ^1^, is recognised as a serious threat to human health ^2, 3^. Impacts on human health include increases in vector-borne diseases, health-related illnesses and mental health conditions ^4– 6^. Paradoxically, the healthcare sector responsible for treating these illnesses contributes to 4.4% to 5.2% of global greenhouse gas emissions ^7, 8^. Healthcare systems have an important role in responding to the challenges of the planetary crisis (e.g. addressing the changing disease burden) and moving towards more sustainable ways of delivering care. Specifically, healthcare professionals have a pivotal role in adapting to, and mitigating against, the health impacts of this crisis.

To undertake this role, healthcare professionals must be equipped with knowledge, attitudes and skills in climate change and sustainability (CC&S). The knowledge, skills and attitudes required of healthcare professionals can be described in different ways, including as a framework of multiple competencies. A competency can be defined as “a learnable, durable, and measurable ability to execute a specific, integrative task that is a part of the full range of tasks that constitute the ... profession. It is a generalized ability that may vary somewhat, depending on the context” ^9^. Competency frameworks outline what an individual is expected to be able to do in their practice ^10^. Alternatively learning outcomes, which are defined as “broad statements of what is achieved and assessed at the end of a course of study” ^11^, may be used. Curricular frameworks include learning outcomes and more detailed information about content and approaches to curriculum delivery.

There have been several calls for the development of competency frameworks in CC&S across healthcare professions ^12^. In response to these calls, organisations have worked to define competencies and learning outcomes both for single healthcare professions and for all healthcare professionals. Examples include the Canadian Federation of Medical Students Health and Environment Adaptive Response Task Force (CFMS HEART) ^13^ and the Sustainable Healthcare Education network ^14^. With this increased body of work, there is a need to identify and synthesize the breadth of competencies, learning outcomes and curricular frameworks that have been developed internationally. The interprofessional nature of contemporary healthcare practice demands that the learning needs of the workforce to respond to the planetary crisis be considered as a whole. Without a comprehensive summary, there is a danger of duplication of efforts and missed opportunities to learn from best practices. A review would also help to identify gaps in the literature and areas for further development.

Previous reviews have aimed to summarise the literature in this area. A review by Parker *et al.* ^15^ aimed to synthesise the literature on competencies in CC&S for all healthcare professionals. The review identified 23 articles conducted before 2018 that outlined suggested competencies for healthcare professionals. All included articles were conducted in four countries in the Global North and 70% of articles related to nursing and medicine. A recent review by Visser *et al.* ^16^ summarised literature on planetary health in medical education only, categorising learning objectives in this area into eight themes as part of the review. The authors noted that most articles were published in 2020 or after and were predominantly conducted in North America and European countries. The authors recognised a limitation of their review as missing literature from other healthcare professions. Given the recent and rapidly-evolving nature of the literature, an up-to-date review focused on all healthcare professions is necessary. Furthermore, these reviews neglected to focus on the rationale and methods for the development of competencies and learning outcomes which would enable insight into their quality.

## Review question

This scoping review will summarise the competencies, curricular frameworks and learning outcomes in climate change and sustainability that have been proposed internationally for healthcare professionals, as well as information on the rationale and processes involved in developing these competencies, frameworks and learning outcomes.

The primary research question is:

What competencies, curricular frameworks and learning outcomes in climate change and sustainability have been proposed internationally for all healthcare professionals?

The secondary research questions are:

1. What are the commonalties and differences across these competencies, frameworks and learning outcomes?
2. What are the rationale and processes involved in developing these competencies, frameworks and learning outcomes?
3. What populations of healthcare professionals have these competencies, frameworks and learning outcomes been developed for?

## Inclusion criteria

The Participants, Concept and Context (PCC) framework was used to develop the research question and to inform the inclusion and exclusion criteria ^17^.

### Participants

Sources relating to healthcare professionals and healthcare students will be included. Healthcare professionals are individuals who “study, diagnose, treat and prevent human illness, injury and other physical and mental impairments in accordance with the needs of the populations they serve” ^18^. This includes medical doctors, nursing and midwifery professionals, dentists and pharmacists. We will include all healthcare professions regulated by Ireland’s multi-profession health and social care regulator, CORU, including “soon to be included” professions. This list of healthcare professions is specified on the CORU website ( www.coru.ie). We will exclude social care workers and social workers from this list, as they do not fall under the definition of healthcare professionals.

### Concept

We will include competencies, curricular frameworks and learning outcomes. We will exclude sources that discuss competencies and learning outcomes without a structure or framework.

### Context

We will include sources in all healthcare and health professions’ education settings. This includes undergraduate, postgraduate and continuing professional education settings.

### Types of sources

The review will consider all study methodologies. Qualitative, quantitative and mixed methods sources will be included in the review. Text and opinion sources will be considered for inclusion in the review. Reports and curriculum documents will also be included.

## Methods

A scoping review was chosen as the most appropriate design for this study. As the review aimed to understand what competencies, curricular frameworks and learning outcomes are in existence and how they have been developed, a scoping review was deemed relevant over alternative review designs ^19^.

The scoping review will be conducted in accordance with the Joanna Briggs Institute (JBI) methodology for scoping reviews ^17^. The Preferred Reporting Items for Systematic Reviews and Meta-Analysis extension for scoping reviews (PRISMA-ScR) will be followed when reporting the review ^20^. The review will follow the six-step methodology proposed by Arksey and O Malley ^21^, with updates by Levac ^22^. These steps are (1) identifying the research question, (2) identifying relevant studies, (3) study selection, (4) charting the data, (5) collating, summarising and reporting the results, and (6) consultation exercise with stakeholders. This scoping review protocol was registered on the Open Science Framework ( https://osf.io/vnx2g). The protocol was reported in line with the best practice guidance and reporting items for the development of scoping review protocols ^23^ (See Appendix 1 in Extended data).

### Search strategy

The search strategy was developed by the research team. A scoping search was conducted in PubMed to identify relevant search terms. The search strategy was developed to encompass terms relating to the three concepts of the research question 1) “healthcare professionals”, 2) “climate change and sustainability” and 3) “competencies”. Free text terms and Medical Subject Headings (MeSH) terms were combined to form the search strategy. The search strategy was tested in Embase and PubMed, then refined and finalised by the research team. The final search strategy for PubMed can be seen in Appendix 2 (See Extended data). Searches will be limited to titles and abstracts.

The following databases will be searched: PubMed, Embase, CINAHL, PsycINFO, SocINDEX, Academic Search Complete, Business Source Complete, British Education Index, Australian Education Index, Scopus and ERIC. The search will be limited to sources from January 2014 to June 2024, as an initial search revealed limited articles of relevance before this time point. Furthermore, a review by Parker *et al.* ^15^ synthesising literature on environmental competencies for healthcare professionals, noted that the majority of included articles were published after 2015. Only sources published in English will be included. Grey literature will also be searched, recognising that competencies, curricular frameworks and learning outcomes may have been published by professional bodies and universities. The grey literature search will comprise a Google search using relevant terms.

### Study/source of evidence selection

The sources identified from the database searched will be imported into Covidence. Covidence in a literature screening tool and has been endorsed by the Joanna Briggs Institute ( www.covidence.org). Duplicates will be removed at this stage. Title and abstract screening will be conducted independently by two reviewers. Potentially relevant sources will be retrieved in full. Following a pilot test on a sample (10%) of the sources, full-text screening against the eligibility criteria will be conducted by two independent reviewers. Any disagreement will be resolved through discussion amongst the reviewers. If a disagreement cannot be resolved, a third reviewer will be consulted. Covidence will record decisions made at this stage. Two reviewers will independently assess the grey literature for inclusion. The eligibility criteria that will be applied when screening can be seen in Table 1. Following full-text review, backward and forward citation tracking will be conducted on the included sources. The results of the selection process will be recorded in a PRISMA Flow Diagram ^24^ and reported in the final scoping review.

**Table 1.** Eligibility criteria.

Inclusion criteria	Exclusion criteria
Competencies, curricular framework and learning outcomes in the area of climate change and sustainability for healthcare professionals and healthcare students.	Not published in English.
Published between January 2014 and June 2024.	Not healthcare students or professionals.
	Sources that discuss competencies or learning outcomes without a structure or framework.
	Sources that reference an established framework without adaptation.
	Insufficient information on competencies or learning outcomes.
	List of topics in a curriculum, without reference to competencies or learning outcomes.

### Data extraction/charting

Data will be extracted from the included sources by two independent reviewers using a data extraction tool. The data extraction tool will be piloted on a sample (10%) of the sources. This will ensure that the extraction process of the two reviewers is consistent. Any disagreements that arise between the reviewers will be resolved through discussion. If the disagreement cannot be resolved, the wider research team will be consulted to assist with the decision. The data extraction tool will be revised throughout the data extraction process to ensure the accurate representation of all sources. Any modifications to the tool will be reported in the scoping review. Where required, authors of sources will be contacted to request missing or additional data. Covidence ( www.covidence.org) and Lumivero NVivo Version 14 ( www.lumivero.com/product/nvivo/) will be used for data extraction. A draft data extraction tool can be seen in Appendix 3 (See Extended data).

### Data analysis and presentation

Presentation of the results will be in the form of a narrative summary and tables. Two reviewers will summarise the findings from the extracted data. Information regarding competencies, domains and learning outcomes will be put into a matrix under descriptive headings. Competencies and learning outcomes will be coded and themed using framework analysis. The lead reviewer will draft the final report, with input from the research team. The review’s rigour will be supported by keeping an audit trail of decisions made throughout the analysis and by triangulation of the findings with the literature and key stakeholders (as described below).

### Consultation exercise

Consultation with key stakeholders in an optional stage in the six-step scoping review process proposed by Arksey and O’Malley ^21^. We will consult with stakeholders throughout the review. The stakeholder group will comprise health professions’ educators, sustainability experts, patient and public representatives and policymakers. Following the full-text screening stage, we will contact key stakeholders by email and ask them to identify sources that they deem relevant to the review. If we have not already identified the source, we will screen it following the steps outlined above. We will also consult with key stakeholders at a meeting to gather feedback on the initial scoping review findings. We will document the outcomes of these communications in the final report. The authors recognise that this is a valuable stage to ensure the search includes all potential data sources and that the relevance of the research will be improved by gathering feedback on the findings.

## Study status

At the time of publication, the review is at the full-text screening stage.

## Discussion

This review will identify and synthesise the available evidence on competencies, curricular frameworks and learning outcomes in climate change and sustainability (CC&S) for healthcare professionals. This review will highlight gaps in the literature and highlight areas for future development. The findings will be used to inform the development of a set of core competencies in CC&S for all health professions. The strengths of this review include the comprehensive search strategy, methodological rigour and consultation with key stakeholders to ensure the relevance and applicability of the findings.

The findings of this review will be published in a peer-reviewed journal. We will disseminate the findings at national and international conferences and at knowledge-sharing events. We will also produce a summary of the findings for our key stakeholders.

## Data Availability

No underlying data are associated with this article. Open Science Framework: Competencies and learning outcomes for healthcare professionals in climate change and sustainability: a scoping review. https://osf.io/2xr7h/ ^25^. This project contains the following extended data: Scoping review protocol extended data.pdf Data are available under the terms of the Creative Commons Attribution 4.0 International Public License.

## Software availability statement

Rayyan ( https://www.rayyan.ai) is a free alternative to Covidence. RQDA ( https://rqda.r-forge.r-project.org/) is a free alternative to Lumivero NVivo.

## References

[1] United Nations Framework Convention on Climate Change: What is the triple planetary crisis? 2022; [updated 2022 Apr 13; cited 2024 Aug 24]. Reference Source

[2] Costello A Abbas M Allen A : Managing the health effects of climate change: *Lancet* and University College London Institute for Global Health Commission. *Lancet.* 2009;373(9676):1693–733. 10.1016/S0140-6736(09)60935-1 19447250

[3] World Health Organisation: COP24 special report: health and climate change. Geneva,2018; [updated 2018 Dec 3; cited 2024 Aug 24]. Reference Source

[4] Vicedo-Cabrera AM Scovronick N Sera F : The burden of heat-related mortality attributable to recent human-induced climate change. *Nat Clim Chang.* 2021;11(6):492–500. 10.1038/s41558-021-01058-x 34221128 PMC7611104

[5] Caminade C McIntyre KM Jones AE : Impact of recent and future climate change on vector-borne diseases. *Ann N Y Acad Sci.* 2019;1436(1):157–173. 10.1111/nyas.13950 30120891 PMC6378404

[6] Rocque RJ Beaudoin C Ndjaboue R : Health effects of climate change: an overview of systematic reviews. *BMJ Open.* 2021;11(6): e046333. 10.1136/bmjopen-2020-046333 34108165 PMC8191619

[7] Health Care Without Harm: Health care's climate footprint.2019; [updated 2019 Oct 3; cited 2024 Jul 23]. Reference Source

[8] Romanello M Di Napoli C Drummond P : The 2022 report of the *Lancet* Countdown on health and climate change: health at the mercy of fossil fuels. *Lancet.* 2022;400(10363):1619–1654. 10.1016/S0140-6736(22)01540-9 36306815 PMC7616806

[9] Ten Cate O : Competency-Based Postgraduate Medical Education: past, present and future. *GMS J Med Educ.* 2017;34(5): Doc69. 10.3205/zma001146 29226237 PMC5704607

[10] Albarqouni L Hoffmann T Straus S : Core competencies in Evidence-Based Practice for health professionals: consensus statement based on a systematic review and delphi survey. *JAMA Netw Open.* 2018;1(2): e180281. 10.1001/jamanetworkopen.2018.0281 30646073

[11] Harden RM : Learning outcomes and instructional objectives: is there a difference? *Med Teach.* 2002;24(2):151–5. 10.1080/0142159022020687 12098434

[12] Ghosh AK Azan A Basu G : Building climate change into medical education: a society of general internal medicine position statement. *J Gen Intern Med.* 2024;39(13):2581–2589. 10.1007/s11606-024-08690-1 38424345 PMC11436550

[13] Canadian Federation of Medical Students Health and Environment Adaptive Response Task Force (CFMS HEART). Planetary Health Educational Competencies.2021; [updated 2021 Dec; cited 2024 Jul 24]. Reference Source

[14] Thompson T Walpole S Braithwaite I : Learning objectives for sustainable health care. *Lancet.* 2014;384(9958):1924–5. 10.1016/S0140-6736(14)62274-1 25435446

[15] Parker G Berta W Shea C : Environmental competencies for healthcare educators and trainees: a scoping review. *Health Educ J.* 2019;79(3):327–45. 10.1177/0017896919886599

[16] Visser EH Oosterveld B Slootweg IA : The development and characteristics of planetary health in medical education: a scoping review. *Acad Med.* 2024. 10.1097/ACM.0000000000005796 38954502

[17] Peters MDJ Marnie C Tricco AC : Updated methodological guidance for the conduct of scoping reviews. *JBI Evid Synth.* 2020;18(10):2119–2126. 10.11124/JBIES-20-00167 33038124

[18] World Health Organization: Transforming and scaling up health professionals’ education and training World Health Organization Guidelines 2013. Geneva,2013; [updated 2013 Oct 1; cited 2024 Aug 24]. Reference Source 26042324

[19] Munn Z Peters MDJ Stern C : Systematic review or scoping review? Guidance for authors when choosing between a systematic or scoping review approach. *BMC Med Res Methodol.* 2018;18(1): 143. 10.1186/s12874-018-0611-x 30453902 PMC6245623

[20] Tricco AC Lillie E Zarin W : PRISMA Extension for Scoping Reviews (PRISMA-ScR): checklist and explanation. *Ann Intern Med.* 2018;169(7):467–473. 10.7326/M18-0850 30178033

[21] Arksey H O'Malley L : Scoping studies: towards a methodological framework. *Int J Soc Res Methodol.* 2005;8(1):19–32. 10.1080/1364557032000119616

[22] Levac D Colquhoun H O'Brien KK : Scoping studies: advancing the methodology. *Implement Sci.* 2010;5(1): 69. 10.1186/1748-5908-5-69 20854677 PMC2954944

[23] Peters MDJ Godfrey C McInerney P : Best practice guidance and reporting items for the development of scoping review protocols. *JBI Evid Synth.* 2022;20(4):953–968. 10.11124/JBIES-21-00242 35102103

[24] Page MJ McKenzie JE Bossuyt PM : The PRISMA 2020 statement: an updated guideline for reporting systematic reviews. *BMJ.* 2021;372:n71. 10.1136/bmj.n71 33782057 PMC8005924

[25] Galvin E Mulcaire R O Brien C : Competencies and learning outcomes for healthcare professionals in climate change and sustainability: a scoping review.2024. 10.17605/osf.io/2xr7h PMC11966091 40181811

