# Supplementary material for: Competencies and learning outcomes for healthcare professionals in climate change and sustainability: A protocol for a scoping review
Source: HRB Open Res. 2024 Oct 16;7:66. [Version 1] doi: 10.12688/hrbopenres.13966.1 (PMC11966091; doi:10.12688/hrbopenres.13966.1)
Supplement: Peer review history

# Reviewer report: Reviewer response for version 1

**DOI:** 10.21956/hrbopenres.15322.r46519

**Published:** 2025-04-02

**Authors:** Ka Yan Hess

**Affiliations:** 1 Oxford Brookes University, Oxford, UK

Thank you for the opportunity to review this much needed work in this important topic. 

**Methods:**

 Please specify if you are using cinahl OR cinahl full text. Why Business source complete?

 Stakeholders - some idea about who please?

 Where appropriate within the text:

 Please define what you meant by competencies, curricular framework and learning outcomes as well as sustainability (because some people mean continuity and others means environmental sustainability). Just be explicit.. 

 Looking forward to see your final work!

Is the study design appropriate for the research question?

Yes

Is the rationale for, and objectives of, the study clearly described?

Yes

Are sufficient details of the methods provided to allow replication by others?

Partly

Are the datasets clearly presented in a useable and accessible format?

Not applicable

Reviewer Expertise:

Allied health profession practice and education; sustainability, planetary health, wellbeing

I confirm that I have read this submission and believe that I have an appropriate level of expertise to confirm that it is of an acceptable scientific standard.

# Reviewer report: Reviewer response for version 1

**DOI:** 10.21956/hrbopenres.15322.r43422

**Published:** 2024-12-12

**Authors:** J H Irlam

**Affiliations:** 1 University of Cape Town, Rondebosch, Western Cape, South Africa

The article outlines a planned scoping review to synthesize existing evidence on competencies, curricular frameworks, and learning outcomes related to climate change and sustainability (CC&S) for healthcare professionals. The protocol addresses the planetary crisis's impact on human health and the healthcare sector's responsibility to both mitigate and adapt to this crisis.

 Rationale and Objectives:

 The healthcare sector contributes significantly to global greenhouse gas emissions while also being tasked with addressing health crises stemming from climate change. The review aims to summarize competencies, curricular frameworks and learning outcomes in climate change and sustainability developed globally for healthcare professionals; identify gaps; and propose areas for improvement.

 Methods:

 The review follows the Joanna Briggs Institute guidelines for scoping reviews and incorporates stakeholder consultation, which is appropriate for the research question. 

 The Participants, Concept and Context (PCC) framework was used to develop the research question and to inform the inclusion and exclusion criteria. The inclusion criteria are clearly stated: the focus is on healthcare professionals (and students), regulated by Ireland’s multi-profession health and social care regulator, CORU, as listed on their website.

 Only sources published in English from 2014 to June 2024 will be included, as an initial search revealed limited earlier articles of relevance. The potential limitations of the language restriction are not mentioned; this should be included in the published report The final search strategy for PubMed is included as an Appendix. Grey literature will also be identified from a Google search using relevant terms, which are not described. These should be included in the final report.

 Data extraction and thematic analysis will be carried out by independent reviewers, with referral to the team where necessary. The data extraction tool will be piloted on a 10% sample of sources.

 In summary, I am satisfied that the protocol is scientifically sound.

 ==============================================

Is the study design appropriate for the research question?

Yes

Is the rationale for, and objectives of, the study clearly described?

Yes

Are sufficient details of the methods provided to allow replication by others?

Yes

Are the datasets clearly presented in a useable and accessible format?

Not applicable

Reviewer Expertise:

Education about Planetary Health and Sustainable Healthcare

I confirm that I have read this submission and believe that I have an appropriate level of expertise to confirm that it is of an acceptable scientific standard.

## References

[1] : Competencies and learning outcomes for healthcare professionals in climate change and sustainability: A protocol for a scoping review. *HRB Open Research* .2024;7: 10.12688/hrbopenres.13966.1 10.12688/hrbopenres.13966.1 PMC1196609140181811
